# Genome Sequencing of Extended-Spectrum β-Lactamase (ESBL)-Producing *Klebsiella pneumoniae* Isolated from Pigs and Abattoir Workers in Cameroon

**DOI:** 10.3389/fmicb.2018.00188

**Published:** 2018-02-09

**Authors:** Luria L. Founou, Raspail C. Founou, Mushal Allam, Arshad Ismail, Cyrille F. Djoko, Sabiha Y. Essack

**Affiliations:** ^1^Antimicrobial Research Unit, College of Health Sciences, University of KwaZulu-Natal, Durban, South Africa; ^2^Department of Food Safety and Environmental Microbiology, Centre of Expertise and Biological Diagnostic of Cameroon, Yaoundé, Cameroon; ^3^Department of Clinical Microbiology, Centre of Expertise and Biological Diagnostic of Cameroon, Yaoundé, Cameroon; ^4^Sequencing Core Facility, National Health Laboratory Service, Johannesburg, South Africa; ^5^Centre for Research and Doctoral Training in Life Science, Health and Environment, The Biotechnology Centre, University of Yaoundé I, Yaoundé, Cameroon; ^6^Metabiota Inc., Yaoundé, Cameroon

**Keywords:** whole genome sequencing, ESBL, *Klebsiella pneumoniae*, Enterobacteriaceae, food chain

## Abstract

**Background and objectives:** Extended-spectrum β-lactamase (ESBL)-producing *Klebsiella pneumoniae* is a serious public health issue globally. In this study, the antibiotic resistance genes, virulence factors, mobile genetic elements, and genetic lineages of circulating ESBL-producing *K. pneumoniae* strains isolated from pigs and humans in Cameroonian abattoirs were investigated using whole genome sequencing (WGS), in order to ascertain zoonotic transmission (viz. from animals to humans and/or vice-versa) in the food chain.

**Methods:** During March–October 2016, 288 nasal and rectal pooled samples from 432 pigs as well as nasal and hand swabs from 82 humans were collected from Cameroon and South Africa. Seven ESBL-producing *K. pneumoniae* circulating in Cameroonian pig abattoirs were selected and their genomic DNA sequenced using an Illumina MiSeq platform. Generated reads were *de novo* assembled using the Qiagen CLC Genomics Workbench and SPAdes. The assembled contigs were annotated using RAST and antibiotic resistance genes, virulence factors, plasmids, and bacteriophages were identified with ResFinder, Virulence Finder, PlasmidFinder, and PHAST, respectively.

**Results:** ESBL-producing *K. pneumoniae* were detected in pigs (34/158; 21.52%) and exposed workers (8/71; 11.26%) in Cameroon only. The circulating *K. pneumoniae* strains were dominated principally by the sequence type (ST) 14 and 39. In addition, the “high-risk” ST307 clone and two novel STs assigned ST2958 and ST2959 were detected. Genomic analysis identified various antibiotic resistance genes associated with resistance to β-lactams, aminoglycosides, fluoroquinolones, macrolide, lincosamide and streptogramins, rifampicin, sulfonamides, trimethoprim, phenicols and tetracycline. None of the ESBL-producing *K. pneumoniae* harbored virulence genes. Intermingled *K. pneumoniae* populations were observed between pig- and human-source within and across abattoirs in the country.

**Conclusion:** Our study shows that ESBL-producing *K. pneumoniae* is actively disseminating in pigs and occupationally exposed workers in Cameroonian pig abattoirs and is probably underestimated in the absence of molecular epidemiological studies. It suggests pigs, abattoir workers and food products as potential reservoirs and sources of zoonotic transmission in Cameroon. Our findings underline the existence of a potential unheeded food safety and public health threat associated with these resistant strains and reinforce the crucial importance of implementing appropriate food safety measures and promoting rational antibiotic use.

## Introduction

*Klebsiella pneumoniae* is an important Gram-negative bacillus associated with several clinical infections in humans (Perovic et al., 2014). Of particular concern is the emergence of extended-spectrum β-lactamase (ESBL) producing *K. pneumoniae* in hospital settings (Perovic et al., 2014) which has considerably increased during the last decade in response to the selection pressure of extensive antibiotic use. This resistant strain is considered a significant public health issue due to the limited therapeutic options and increased morbidity and mortality associated with it (World Health Organization [WHO], 2017). However, the concern of ESBL-producing *K. pneumoniae* goes beyond healthcare settings to affect various ecological niches including (food) animals, food products, soil and wastewater. In fact, humans may become colonized or infected by ESBL-producing *K. pneumoniae* upon contact with blood, saliva, feces and urine of ESBL carrier animals or consumption of contaminated water or food products (Founou et al., 2016).

The most frequent and clinically relevant ESBL genes belong to CTX-M, TEM, and SHV families, with CTX-M enzymes emerging as the predominant type. CTX-M is divided into five groups namely CTX-M-1, CTX-M-2, CTX-M-8, CTX-M-9, and CTX-M-25 according to their amino-acid identities (Perovic et al., 2014). *K. pneumoniae* commonly produces all three groups of enzymes but the latest public health concern has been the emergence of carbapenemase-producing *K. pneumoniae* and colistin-resistant *K. pneumoniae* (Hudson et al., 2014; Perovic et al., 2016). These resistance genes are generally carried on mobile genetic elements (MGEs) facilitating their dissemination within and between bacterial species (Founou et al., 2016). The presence of MGEs likely increases the proportion of serious difficult-to-treat *K. pneumoniae* infections.

The true prevalence of ESBL is not well-known in Africa and probably underestimated because of the paucity of studies in human health, animal health, and the food chain on the continent. Nevertheless, some studies, have confirmed the global distribution and the high prevalence of ESBL-producing *K. pneumoniae* on the continent, albeit focusing on human health sector and ignoring the animal one (Gangoue-Pieboji et al., 2005; Breurec et al., 2013; Belbel et al., 2014; Jacobson et al., 2015; Lyonga et al., 2015; Nzalie et al., 2016). For example, a multicenter study, detected ESBL-producing *K. pneumoniae* in public hospitals in Abidjan, Casablanca, Yaoundé, and Antananarivo with prevalence ranging from 9 to 16%. The sequence type (ST) 15 and ST 11 were the most predominant *K. pneumoniae* clonal lineages (Breurec et al., 2013). During a national sentinel site surveillance of resistant *K. pneumoniae*, Perovic et al. (2014) reported a 68.3% prevalence of ESBL-producing *K. pneumoniae* in clinical samples from 2010 to 2012. Magoué et al. (2013) reported a 22.15% prevalence of fecal ESBL-producing *K. pneumoniae* carriage in Ngaoundere, Cameroon, and showed the high endemicity of CTX-M-15 producers in the country. However, the genetic background of these ESBL producers were only investigated in very few of these studies. The rare African studies reporting ESBL-producing *K. pneumoniae* in food products emanated from Benin and Sudan where ESBL-producing *K. pneumoniae* were detected in 5.77% of vegetables (Moussé et al., 2016) and 62% of raw milk (Badri et al., 2017), respectively.

In Cameroon, where antibiotics are used without restriction not only in the healthcare sector but also in the food production industry, the epidemiology of antibiotic resistant bacteria in food animals and associated public health implications is neglected. This study investigated the antibiotic resistance genes, virulence factors, MGEs and genetic lineages of circulating ESBL-producing *K. pneumoniae* strains isolated from pigs and exposed workers in Cameroonian abattoirs using whole genome sequencing (WGS), to ascertain zoonotic transmission (viz. from animals to humans and/or vice-versa) of ESBL-producing *K. pneumoniae* in the food chain.

## Materials and Methods

### Ethical Approvals

Ethical approvals were obtained from the Biomedical Research Ethics Committee (Ref. BE365/15) and Animal Research Ethics Committee (Ref. AREC/091/015D) of the University of KwaZulu-Natal as well as from the National Ethics Committee for Research in Human Health of Cameroon (Ref. 2016/01/684/CE/CNERSH/SP) prior to the implementation of the study. Ministerial approvals were also obtained from the Cameroonian Ministry of Livestock, Fisheries and Animal Industries (Ref. 061/L/MINEPIA/SG/DREPIA/CE) and Ministry of Scientific Research and Innovation (Ref. 015/MINRESI/B00/C00/C10/C14).

### Study Design and Bacterial Isolates

From March to October 2016, a multi-center study was carried out in three slaughterhouses/markets in Cameroon and two abattoirs in South Africa, encoded for ethical reasons as SH001, SH002, SH003 and SH004 and SH005, respectively (unpublished data). Three individual samples were pooled to yield 144 nasal and 144 rectal pools representing 432 original nasal and rectal samples, respectively, collected from 432 pigs. A total of 288 swabs from the 144 nasal and 144 rectal pools constituted the final sample. Nasal and hand swabs were also collected from 82 humans in both Cameroon and South Africa. All samples were cultured on MacConkey agar supplemented with 2 mg/L cefotaxime and incubated for 18–24 h at 37°C in normal atmosphere (unpublished data). All putative ESBL-producers, were subjected to Gram staining, and the catalase and oxidase tests for phenotypic characterization of the isolates to the genus level. The isolates were thereafter phenotypically confirmed using the VITEK 2 system. The strains sequenced in this study were isolated from four pig pooled samples (PN030E4, PN089E1, PN085E1IA, and PR042E3) and three exposed workers (HH510E2I, HH517E1II, and HN523E1II) in Cameroon (unpublished data). The pig isolates, PN30E4 and PR042E3, originated from the same abattoir (SH001), although the former was collected from the nares and the latter from rectum. Likewise, the strains, PN089E1 and PN085E1IA, were both collected from the nares of pigs processed in abattoir SH002. The human strains, HH510E2I, HH517E1II, and HN523E1II, originated from three different abattoirs, SH001, SH002, and SH003, respectively, with HH510E2I and HH517E1II being collected from hands and HN523E1II from the nares. These strains were identified in a previous study, to be highly closely related via enterobacterial-repetitive-polymerase chain reaction (ERIC-PCR) analysis where they were grouped into five clusters (unpublished data). Given that we aimed to highlight and provide evidence of zoonotic transmission (i.e., from animals to humans and vice-versa) of ESBL-*K. pneumoniae* in the food chain, within each generated cluster, only representative isolates of intermingled strains (i.e., strains isolated from animal and/or human of the same abattoir, having high genetic relationship with those from another abattoir) were considered for WGS.

### Screening for ESBL

The standard double disk synergy test (DDST), using cefotaxime and ceftazidime, alone and in combination with clavulanic acid as recommended by the Clinical Laboratory and Standards Institute (CLSI) was performed for ESBL screening. An increase in size of the inhibition zone of more than 5 mm in the presence of clavulanic acid was regarded as proof for ESBL production (Clinical and Laboratory Standards Institute [CLSI], 2016).

### Antimicrobial Susceptibility Testing

Antimicrobial susceptibility testing was performed to determine the resistance patterns of the selected strains. Ampicillin, amoxicillin + clavulanic acid, cefuroxime, cefotaxime, ceftazidime, cefoxitin, cefepime, ertapenem, imipenem, meropenem, gentamicin, amikacin, ciprofloxacin, tigecycline, nitrofurantoin, piperacillin/tazobactam, colistin and trimethoprim-sulfamethoxazole, were tested using Vitek^®^ 2 System and Vitek^®^ 2 Gram Negative Susceptibility card (AST-N255) (BioMérieux, Marcy l’Etoile, France). The results were interpreted according to the CLSI guidelines (Clinical and Laboratory Standards Institute [CLSI], 2016) with the exception of colistin, amoxicillin + clavulanic acid, piperacillin/tazobactam, amikacin that were based on EUCAST breakpoints (The European Committee on Antimicrobial Susceptibility Testing [EUCAST], 2016) with *E. coli* ATCC 25922 and *K. pneumoniae* ATCC700603 being used as controls.

### DNA Isolation

Genomic DNA (gDNA) was extracted using GenElute^®^ bacterial genomic DNA kit (Sigma–Aldrich, St. Louis, MO, United States) according to the manufacturer’s instructions. The quantification of extracted gDNA was determined on a NanoDrop spectrophotometer with verification by agarose gel electrophoresis and fluorimetric analysis (Qubit^®^).

### Genotypic Relatedness Determination of ESBL-Producing *K. pneumoniae*

To establish the link of selected ESBL-producing *K. pneumoniae* strains from animals and humans within and between abattoirs, ERIC-PCR was performed with primers ERIC 1 5′-ATG TAA GCT CCT GGG GAT TCA C-3′ and ERIC2 5′-AAG TAA GTG ACT GGG GTG AGC G-3′ (Versalovic et al., 1991). Reactions were carried out in a 10 μl final solution containing 0.1 μl of each primer (100 μM), 5 μl Dream*Taq* Green Polymerase Master Mix 2× (Thermo Fisher Scientific, South Africa), 2.8 μl nuclease free water and 2 μl DNA template and run in an Applied Biosystems 2720 programmable thermal cycler (Thermo Fisher Scientific, South Africa) with the following protocol: initial denaturation at 94°C for 3 min, 30 cycles consisting of a denaturation step at 94°C for 30 s, annealing at 50°C for 1 min, extension at 65°C for 8 min, a final extension step at 65°C for 16 min and final storage at 4°C. ERIC profiles were digitized for analysis using Bionumerics software (version 7.6, Applied Maths, Austin, TX, United States). The similarity between each strain was determined from the homology matrix using Dice coefficient and dendrograms constructed using the algorithm Unweighted Pair-Group Method (UPGMA).

### Genome Sequencing

Multiplexed paired-end libraries (2 × 300 bp) were prepared using the Nextera XT DNA sample preparation kit (Illumina, San Diego, CA, United States) and sequences determined on an Illumina MiSeq platform with 100× coverage at the National Institute of Communicable Diseases Sequencing Core Facility, South Africa.

### Genome Assembly

The resulting raw reads were checked for quality, trimmed and *de novo* assembled into contigs using CLC Genomics Workbench version 10 (CLC, Bio-QIAGEN, Aarhus, Denmark) and SPAdes version 3.11 (Bankevich et al., 2012) to overrule any inherent shortfalls from both assemblers.

### Genome Analysis

The *de novo* assembled reads were uploaded in GenBank and annotated using NCBI prokaryotic genome annotation pipeline and RAST 2.0 server^1^ (Aziz et al., 2008) which identified encoding proteins, rRNA and tRNA, assigned functions to the genes and predicted subsystems represented in the genome. The bacterial analysis pipeline of GoSeqIt tool was also used to annotate and identify known acquired antibiotic resistant genes via ResFinder (Zankari et al., 2012), virulence factors [including capsular polysaccharide (CPS), lipopolysaccharide (LPS), adhesin, long polar fimbriae (lpfA), increased serum survival (iss), enterobactin siderophore receptor protein (iroN), biofilm, lipase, ABC transporter protein MchF, and gelatinase] using VirulenceFinder (Joensen et al., 2014) and MGEs through PlasmidFinder (Carattoli et al., 2014). The RAST SEED viewer was used to identify the presence of transposases and integrons flanking the β-lactamase genes (Overbeek et al., 2014). PHAge Search Tool (PHAST) server was used for the identification, annotation, and visualization of prophage sequences (Zhou et al., 2011).

### Multilocus Sequence Typing (MLST)

*In silico* MLST-analyses was performed using the scheme of Diancourt et al. (2005), which consider allelic variation amongst seven housekeeping genes (*gapa*, *infb*, *mdh*, *pgi*, *phoe*, *rpob*, and *tonb*) to assign STs. WGS data were used to generate a *K. pneumoniae* MLST assignment for each isolate with new or unknown STs being sent for curation at the *Klebsiella pneumoniae* MLST database at the Pasteur Institute^2^.

### Whole Genome Phylogenetic Analyses

The assembled contigs were aligned against the complete genome of *K. pneumoniae* KPN528 (CP020853) using the progressive Mauve genome alignment package version 2.3.1. Study genomes were contextualized against a collection of five relevant assembled *K. pneumoniae* genomes (accession numbers AYQE00000000, CP006918, CP020853, CP020847, CP020841) showing high similarity in terms of resistance genes and STs. In addition, only the complete *K. pneumoniae* genomes released 2015 onward were selected to ensure that the analysis depicts current evolution of ESBL-producing *K. pneumoniae*.

Phylogenetic analysis was based on the core genomes and performed using the Rapid large-scale prokaryote pan genome analysis (Roary) (Page et al., 2015). The maximum likelihood phylogenetic tree encompassing, country, population, sample type metadata and MLST type was generated, edited and visualized using FastTree version 2.1.7^3^. In addition, the contigs were mapped against the complete genome of *K. pneumoniae* KPN528 (CP020853) for visualization of the genomic organization.

### Nucleotide Sequence Accession Number

This whole-genome shotgun project PRJNA412434 of *K. pneumoniae* strains PN030E4, HH510E2I, HN523E1II, PN089E1, PR042E3, HH517E1II, and PN085E1IA has been deposited at DDBJ/EMBL/GenBank under accession numbers PDVM00000000, PDVF00000000, PDVG00000000, PDVC00000000, PDVE00000000, PDVU00000000, and PDVD00000000, respectively. The versions described in this paper are the versions, PDVM00000000.1, PDVF00000000.1, PDVG00000000.1, PDVC00000000.1, PDVE00000000.1, PDVU00000000.1, and PDVD00000000.1, respectively.

## Results

### Phenotypic Analyses

Out of the 144 pooled nasal samples (three nasal swabs each) and 144 pooled rectal samples (three rectal swabs each) taken from 432 pigs in Cameroon and South Africa, ESBL-producing *Enterobacteriaceae* were detected in 108/144 (75%) and 102/144 (71%) nasal and rectal pools, respectively (unpublished data). Carriage of ESBL-producing *Enterobacteriaceae* was observed in Cameroonian workers only. Multiple colonies (up to 10) were isolated from both populations and countries, but due to financial constraints, post-stratification allowed the selection of 158 ESBL-PE in pigs and 71 in exposed workers, for phenotypic analysis (unpublished data). Out of these, ESBL-producing *K. pneumoniae* were only detected in Cameroon with 21.52% (34/158) and 11.26% (8/71) detected in pigs and exposed workers, respectively (unpublished data). None of the South African pig or human samples were positive for ESBL-producing *K. pneumoniae*. **Table 1** summarizes relevant population data, specimen source, phenotypic, and genotypic characteristics for these isolates. All isolates displayed reduced susceptibility to the amino-penicillins, cephalosporins, trimethoprim-sulfamethoxazole, with various resistance to gentamicin (*n* = 5; 71%), ciprofloxacin (*n* = 1; 14%) and nitrofurantoin (*n* = 1; 14%). The antimicrobial susceptibility results of the ESBL-producing *K. pneumoniae* isolates are summarized in **Table 2**, with the resistance observed being corroborated with WGS analyses.

**Table 1:** Summary of population, sample type, phenotypic and genotypic characteristics of ESBL-producing *K. pneumoniae* isolates.

Isolate name	Host	Sample type	Abattoir	Antibiotic resistance genes	Plasmids	MLST
PN030E4	Pig	Nasal swab	SH001	*str*A, *str*B, *bla*_TEM-116_, *bla*_SHV -28_, *bla*_CTX-M-15_, *oqx*A, *oqx*B, *Qnr*B1, *fos*A, sul1, sul2, tet(A), dfrA15	ColRNAI, IncFIB(K), IncFIA(HI1), IncY	ST14
HN523E1II	Human	Nasal swab	SH003	*str*A, *str*B, *bla*_TEM-116_, *bla*_SHV -28_, *bla*_CTX-M-15_, *oqx*A, *oqx*B, *Qnr*B1, *fos*A, sul1, dfrA15	ColRNAI, IncFIB(K), IncFIA(HI1)	ST14
HH517E1II	Human	Hand swab	SH002	aac(3)-IIa, *aad*A1, *bla*_TEM-1B_, *bla*_SHV -11_, *bla*_CTX-M-15_, *bla*_SCO-1_, *oqx*A, *oqx*B, *fos*A, sul1, *tet*(A), *dfr*A15	ColRNAI, IncFIB(K), IncHI1B	ST39
PN085E1IA	Pig	Nasal swab	SH002	aac(3)-IIa, *aad*A1, strA, strB, *bla*_TEM-1B_, *bla*_SHV -27_, *bla*_SCO-1_, *bla*_CTX-M-15_, *oqx*A, *oqx*B, *fos*A, *cat*A1, *sul*1, *sul*2, *tet*(A), *dfr*A15	ColRNAI, IncFIB(K), IncFII(K), FIA(pBK30683)	ST39
PR042E3	Pig	Rectal swab	SH001	aac(3)-IIa, *aad*A1, *str*A, *str*B, *bla*_TEM-1B_, *bla*_SHV -1_, *bla*_CTX-M-15_, *oqx*A, *oqx*B, *fos*A, *sul*1, *tet*(A), *dfr*A15	ColRNAI, IncFIB(K)	ST2958
PN089E1	Pig	Nasal swab	SH002	aac(3)-IIa, *aad*A1, *str*A, *str*B, *bla*_TEM-1B_, *bla*_SHV -27_, *bla*_SCO-1_, *bla*_CTX-M-15_, *oqx*A, *oqx*B, *fos*A, *mph*(A), *cat*A1, *sul*1, *sul*2, *tet*(A), *dfr*A15	ColRNAI, IncFIB(K), IncR, ColE10	ST2959
HH510E2I	Human	Hand swab	SH001	aac(6’)-Ib, *str*A, *str*B, aac(3)-IId, aac(6’)Ib-cr^∗^, *aad*A1, *aad*A16, *bla*_OXA-9_, *bla*_LEN-12_, *bla*_SHV -134_, *bla*_TEM-1A_, *oqx*A, *oqx*B, *fos*A, *cat*A2, *ARR*-3, *sul*1, *sul*2, *tet*(D), *dfr*A27	ColRNAI, IncR, FIA(pBK30683)	ST307


**^∗^aac(6’)Ib-cr: confers concomitant resistance to aminoglycosides and fluoroquinolones*.*

**Table 2:** Antimicrobial susceptibility results of selected β-lactam and non-β-lactam antibiotics for individual ESBL-producing *K. pneumoniae* isolates.

Isolate name	β-lactam antibiotics										Non-β-lactam antibiotics							Antibiotic resistance genes
			
	AMP	AMC	TZP	CXM	CTX	CAZ	FEP	ETP	MEM	IMP	GEN	AN	CIP	TGC	FT	CS	TMP/SXT	
HH510E2I	R	R	S	R	R	R	S	S	S	S	R	R	R	S	S	S	R	*aac(6’)-Ib, strA, strB, aac(3)-IId, aac(6’)Ib-cr, aadA1, aadA16, bla_OXA-9_, bla_LEN-12_, bla_SHV -134_, bla_TEM-1A_, oqxA, oqxB, fosA, catA2, ARR-3, sul1, sul2, tet(D), dfrA27*
HH517E1II	R	R	S	R	R	I	S	S	S	S	R	S	S	S	R	S	R	*aac(3)-IIa, aadA1, bla_TEM-1B_, bla_SHV -11_, bla_CTX-M-15_, bla_SCO-1_, oqxA, oqxB, fosA, sul1, tet(A), dfrA15*
PN085E1IA	R	S	S	R	R	R	S	S	S	S	R	S	S	S	S	S	R	*aac(3)-IIa, aadA1, strA, strB, bla_TEM-1B_, bla_SHV -27_, bla_SCO-1_, bla_CTX-M-15_, oqxA, oqxB, fosA, catA1, sul1, sul2, tet(A), dfrA15*
PR042E3	R	S	S	R	R	S	S	S	S	S	R	S	S	S	S	S	R	*aac(3)-IIa, aadA1, strA, strB, bla_TEM-1B_, bla_SHV -1_, bla_CTX-M-15_, oqxA, oqxB, fosA, sul1, tet(A), dfrA15*
PN089E1	R	S	S	R	R	R	S	S	S	S	R	S	S	S	S	S	R	*aac(3)-IIa, aadA1, strA, strB, bla_TEM-1B_, bla_SHV -27_, bla_SCO-1_, bla_CTX-M-15_, oqxA, oqxB, fosA, mph(A), catA1, sul1, sul2, tet(A), dfrA15*
PN030E4	R	S	S	R	R	R	S	S	S	S	S	S	S	S	S	S	R	*strA, strB, bla_TEM-116,_ bla_SHV -28_, bla_CTX-M-15_, oqxA, oqxB, QnrB1, fosA, sul1, sul2, tet(A), dfrA15*
HN523E1II	R	S	S	R	R	R	S	S	S	S	S	S	S	S	I	S	R	*strA, strB, bla_TEM-116,_ bla_SHV -28_, bla_CTX-M-15_, oqxA, oqxB, QnrB1, fosA, sul1, dfrA15*


**AMP, ampicillin; AMC, amoxicillin-clavulanic acid; TZP, piperacillin-tazobactam; CXM, cefuroxime; CTX, cefotaxime; CAZ, ceftazidime; FEP, cefepime; ETP, ertapenem; MEM, meropenem; IMP, imipenem; GEN, gentamicin; AN, amikacin; CIP, ciprofloxacin; TGC, tigecycline; FT, nitrofurantoin; CS, colistin; TMP/SXT, trimethoprim-sulfamethoxazole; S, susceptible; I, intermediate; R, resistant*.*

### Genotypic Analyses

All isolates carried sulfonamide (*sul*1), fosfomycin (*fos*A) and quinolone (*oqx*A and *oqx*B) resistance genes. Various β-lactamase encoding determinants were detected with *bla*_CTX-M-15_ (86%), *bla*_TEM-1B_ (57%) and *bla*_SCO-1_ (43%) being the most prevalent. Four (PN089E1, PN085E1IA, HH517E1II, and HH510E2II) and three (PR042E3, PN030E4, and HN523E1II) isolates concomitantly harbored four and three β-lactamase encoding genes. Likewise, six (86%) strains harbored *dfr*A15 gene responsible for trimethoprim resistance, while co-presence of *str*A and *str*B encoding for aminoglycoside resistance was observed in five (71%) isolates as was the *tet*(A) gene responsible for tetracycline resistance. Interestingly, *bla*_OXA-9_, *bla*_LEN12_, *bla*_SHV -134_, *bla*_TEM-1A_ were observed in the unique *K. pneumoniae* ST307 strain as were *aac(6’)Ib-cr*, *cat*A2, *sul*2, *tet*(D), *drf*A27 and *ARR*-3 encoding resistance to aminoglycosides, phenicols, sulfonamides, tetracycline, trimethoprim and rifampicin, respectively. Similarly, the β-lactamase genes *bla*_TEM-116_ and *bla*_SHV -28_, and plasmid mediated quinolone resistance (PMQR) genes *Qnr*B1 were only identified in the two *K. pneumoniae* ST14 strains. None of the *K. pneumoniae* isolates harbored virulence genes.

### Multilocus Sequence Typing

*In silico* MLST-analyses revealed that *K. pneumoniae* strains belonging to five different STs, namely ST14 (*n* = 2), ST39 (*n* = 2), a single-locus variant ST307 and two new sequences. The two *K. pneumoniae* ST14 strains were isolated from a pig pooled nasal sample (PN30E4) and a human nasal swab (HN523E1II) located in two different abattoirs, SH001 and SH003, respectively (**Table 1**). The *K. pneumoniae* ST39 strains, HH517E1II, and PN085E1IA, were also detected from an exposed worker and pooled nasal samples but both from the same abattoir SH002. In our collection of seven ESBL-producing *K. pneumoniae* isolates, two strains, PR042E3 and PN089E1 isolated from pigs in two different abattoirs (SH001 and SH002), had a novel combination of known *K. pneumoniae* MLST alleles, that were assigned as ST2958 (*n* = 1) and ST2959 (*n* = 1), respectively. The sole *K. pneumoniae* ST307 strain was isolated from hand of an exposed worker and harbored a total of 22 resistance determinants encoding resistance to nine antibiotic classes.

### Phylogenetic Analysis

**FIGURE 1:**
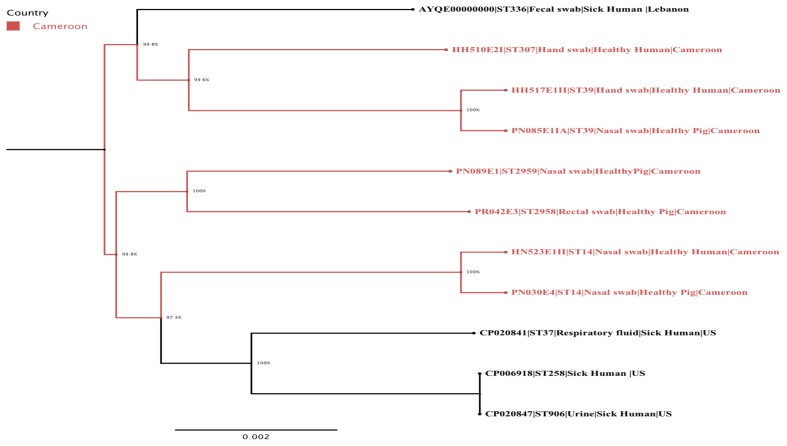
A maximum-likelihood phylogenetic tree of ESBL-producing *K. pneumoniae* isolates generated using FastTree version 2.1.7. The Cameroonian isolates are colored in red. The following information is also provided for each isolate: name/reference, sequence type, type of sample, population, and country.

**FIGURE 2:**
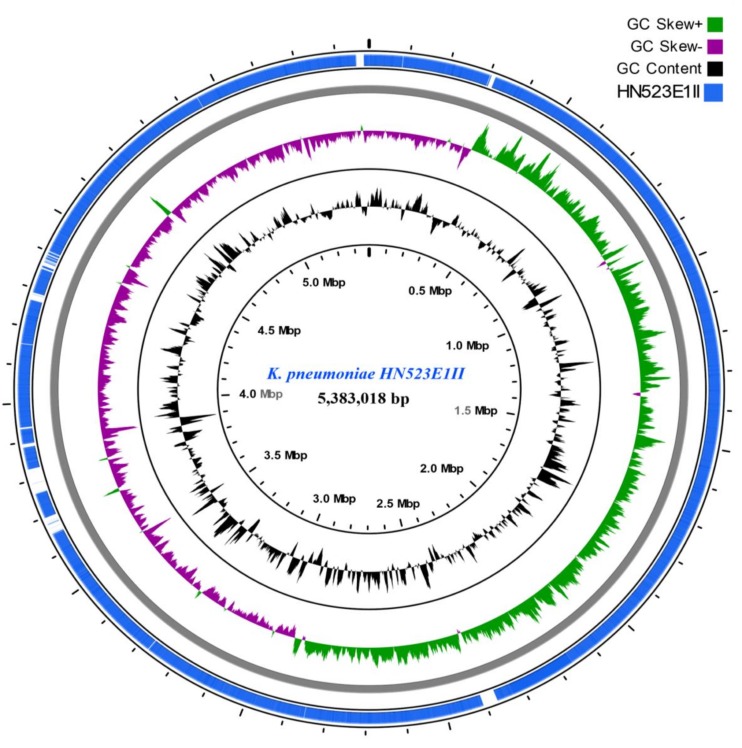
ESBL-producing *K. pneumoniae* HN523E1II ring representation using CGView Server V 1.0 (Grant et al., 2012). The inner ring displays the percent of identity comparing *K. pneumoniae* HN523E1II and the complete genome of *K. pneumoniae* KPN528 (CP020853). The two next (inner) rings display the GC content and GC skew, respectively. The last outer ring indicates the genome *K. pneumoniae* HN523E1II.

The phylogenetic analysis confirmed the intermingled reservoir of ESBL-producing *K. pneumoniae* strains and revealed that our strains fall within two clades of international *K. pneumoniae* isolates. **Figure 1** demonstrates considerable similarity between our collection of *K. pneumoniae* ST14 and three strains CP020841 (ST37), CP006918 (ST258), and CP020847 (ST906), all isolated from clinically ill humans in United States. Similarly, the *K. pneumoniae* ST39 strains isolated from nares of healthy pig (PN085E1IA) and the hand of healthy human (HH517E1II) in abattoir SH002, were closely related *K. pneumoniae* ST307 (HH510E2I) isolated from hand of a human in SH001 and a *K. pneumoniae* ST336 isolated in clinically ill patient in Lebanon (**Figure 1**). **Figure 2** shows the genomic organization of the ESBL-producing *K. pneumoniae* HN523E1II mapped against the complete genome of *K. pneumoniae* KPN528 (CP020853).

### Detection of Plasmids and Phage-Associated Regions

PlasmidFinder revealed that the colRNAI plasmid replicon type was identified in all strains whereas the IncFIB(K) plasmid incompatibility group was detected in six (86%) isolates. The two *K. pneumoniae* ST14 strains, PN030E4 and HN523E1II, concomitantly harbored two colRNAI plasmid replicon types as well as two IncFIA(HI1) and IncFIB(K) plasmid incompatibility groups, with the strain PN030E4 additionally harboring the IncY plasmid. Likewise, the *K. pneumoniae* ST307 harbored two colRNAI replicons as well as FIA (pBK30683) and IncR plasmid incompatibility groups while the *K. pneumoniae* ST 39 carried four colRNAI replicons along with IncHI1B and IncFIB(K) plasmid incompatibility groups. *In silico* plasmid MLST-analyses assigned the IncF plasmid incompatibility group as belonging to various STs including [K1:A13-like:B-], [K-:A10-like:B-], [K-:A13:B-] while the IncH plasmid belonged to a non-typeable ST.

**FIGURE 3:**
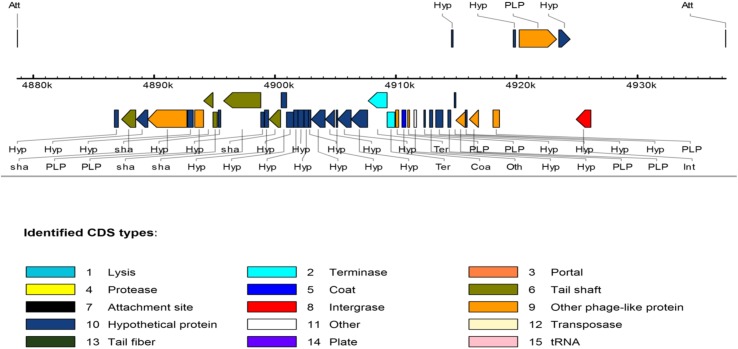
Linear view of the prophage Escher HK639 isolated from the *K. pneumoniae* ST14 (HN523E1II). Putative genes are colored according to the predicted functions of their products.

With regard to the phage-associated regions, all strains hosted at least one intact bacteriophage (**Table 3**). Shigel SfII, pseudo JBD44, Entero lato and Escher HK639 were the predominant intact bacteriophages. Six phage regions were identified in one *K. pneumoniae* ST14 (HN523E1II) isolated from hand of a worker using PHAST algorithm, while the other ST14 (PN030E4) and one ST39 (PN085E1IA) observed in pigs hosted three phage regions each. **Figure 3** shows the genomic structure of the phage Escher HK639 in the *K. pneumoniae* ST14 (HN523E1II).

**Table 3:** Distribution of intact prophage regions among the ESBL-producing *K. pneumoniae* strains.

Isolate name	Region^a^	Length^b^ (kb)	No CDS	GC%	Phage (hit genes count)^c^
HH510E2I	1	37.6	47	51.42	Salmon 103203 sal5 (13)
HH517E1II	1	39.3	52	52.13	Salmon 64795 sal3 (15)
HN523E1II	1	30.3	37	51.02	Entero mEp390 (6)
	2	17.1	20	48.37	Entero lato (7)
	3	27.6	12	53.30	Shigel SfII (8)
	4	17.4	26	54.84	Shigel SfII (18)
	5	58.6	44	53.75	Escher HK639 (18)
	6	29.8	35	51.86	Entero c 1 (9)
PN030E4	1	29.6	35	51.54	Brucel BiPBO1 (6)
	2	23	28	53.03	Shigel SfII (20)
	3	48.6	49	53.27	Escher HK639 (19)
PN085E1IA	1	27.4	28	52.12	Pseudo JBD44 (9)
	2	31.4	25	52.93	Entero c 1 (12)
	3	33.1	12	49.24	Entero lato (7)
PR089E1	1	47.6	41	50.80	Salmon 118970 sal3 (11)
	2	27	34	55.25	Salmon Fels 2 (22)
PR042E3	1	53.1	57	52.17	Pseudo JBD44 (13)


*^*a*^ Intact prophage region; ^*b*^ Region length of intact prophage; ^*c*^ Phage with the highest number of CDS in the region and the number of gene counts in brackets*.

## Discussion

In this study, antibiotic resistance genes, virulence factors, MGEs and genetic lineages of seven circulating and closely related ESBL-producing *K. pneumoniae* strains isolated from pigs and occupationally exposed workers in Cameroonian abattoirs were investigated using WGS.

ESBL-producing *K. pneumoniae* have been observed at diverse rates in clinical samples in the Ivory Coast (16%), Morocco (13%), Cameroon (10%) and Madagascar (9%) (Breurec et al., 2013). They were also responsible of community-acquired urinary tract infections in a Cameroonian city with an incidence of 16.4% (Nzalie et al., 2016). Our study, similarly revealed ESBL-producing *K. pneumoniae* strains in pigs (27.64%) and exposed workers (16.66%) in Cameroon as the first report ESBL-producing *K. pneumoniae* isolates in food animals and occupationally exposed workers in the country. The ESBL-producing *K. pneumoniae* strains were mainly circulating in two clonal lineages since four out of seven isolated strains belonged to the ST14 (*n* = 2) and ST39 (*n* = 2). To the best of our knowledge, this is the first evidence of a porcine ESBL-producing *K. pneumoniae* reservoir in this country. Moreover, the first reports of *bla*_CTX-M-15_, *bla*_TEM-1B_, *bla*_SHV -11_, *bla*_SCO-1_ producing *K. pneumoniae* ST39 and *bla*_CTX-M-15_, *bla*_TEM-116_, *bla*_SHV -28_ producing *K. pneumoniae* ST14 in pigs and humans in Cameroon are presented here.

The *K. pneumoniae* ST14 isolates were found to be resistant to ampicillin, cefuroxime, cefuroxime-axetil, cefotaxime, ceftazidime and trimethoprim-sulfamethoxazole. This resistance phenotype was corroborated by the identification of the CTX-M-15, SHV-28, and TEM-116 genes by WGS which also elucidated with multiple resistant determinants to non-β-lactam antibiotics, notably the aminoglycoside resistant genes (*str*A, *str*B), plasmid-mediated quinolone resistance genes (*Qnr*B1, *oqx*A, *oqx*B), fosfomycin resistant gene (*fos*A), and sulfonamide resistant gene (sul1 and sul2) which were not phenotypically evident. Although the two *K. pneumoniae* ST39 isolates displayed similar phenotypic profiles, these were attributed to different resistance gene permutations. For example, the pig strain PN085E1IA harbored genes encoding for aminoglycoside [aad1, aac(3)-IIa], fluoroquinolones (*oqx*A, *oqx*B), fosfomycin (*fos*A), tetracycline [*tet*(A)], trimethoprim (*dfr*A15) and sulfonamide (*sul*1, *sul*2) resistance alongside blaCTX-M-15, *bla*_TEM-1B_ and *bla*_SHV -1_ while *aad*1, *aac*(3)-IIa, *oqx*A, *oqx*B, fosA, *sul*1, *tet*(A), *dfr*A15, *bla*_TEM-1B_, *bla*_CTX-M-15_, *bla*_SCO-1_, *bla*_SHV -11_ were observed in the human strain HH517E1II. CTX-M-15 detection is consistent with a multicenter study conducted in five African (including Yaoundé) and two Vietnamese towns where it was detected in 74% of isolates and was the predominant ESBL among the African isolates (Breurec et al., 2013). This study further reported the predominance of *Qnr*B determinant among the African strains (Breurec et al., 2013). In addition, CTX-M-15-producing *K. pneumoniae* hosted on a plasmid has already been reported in patients with clinical urinary tract infections in Cameroon (Gangoue-Pieboji et al., 2005), confirming the widespread dissemination of this ESBL type. The preponderance of CTX-M-15 as the main ESBL genes (85.71%) in our *K. pneumoniae* isolates confirms that CTX-M-15 is currently the most largely distributed CTX-M enzyme worldwide. The potential role of *K. pneumoniae* as a reservoir for β-lactam and non-β-lactam resistance determinants is a major concern in countries with inadequate antibiotic resistance (ABR) surveillance, prevention and containment measures such as Cameroon. Key factors favoring the emergence and spread of ABR in the food chain in the country include irrational antibiotic use on farms, poor sanitary and feeding practices, sub-optimal transport conditions, lack of veterinarian control, inadequate diagnostic facilities, substandard quality of antibiotics, lack of antimicrobial monitoring and poor biosecurity measures (Ndebi et al., 2009).

Our study showed that both ST14 and ST39 demonstrated overlap and intermingled populations between pig- and human-sources within and across abattoirs (**Figure 1**). Specifically, the *K. pneumoniae* ST14 strains colonized nares of both human and pigs located in two different abattoirs (SH001 and SH003) whereas *K. pneumoniae* ST39 was detected in nares of pigs and the hand of a worker present in the same abattoir, SH002 (**Figure 1**). This could be associated with neglected hygienic practices prevailing during production, transport, storage and/or retail stages. The virtual absence of physical barriers between community and healthcare settings in this country along with poverty and limited education may also be important contributory factors.

The ST14 and ST39 clonal lineages are a major cause of nosocomial infections and outbreak situations around the world, although their evolutionary emergence is somewhat poorly documented in developing countries. In fact, OXA-181-producing *K. pneumoniae* ST14 was detected in South Africa where it was responsible of an outbreak of among hospitalized patients in a tertiary hospital (Jacobson et al., 2015), whereas, a multidrug resistant and biofilm producing *K. pneumoniae* strain belonging to the ST14 was detected in India also at tertiary care (Rafiq et al., 2016). Similarly, the *K. pneumoniae* ST39 was responsible for an outbreak in a pediatric hospital in Algeria (Belbel et al., 2014). The isolation of these STs always urged the implementation of stringent infection and control measures and ongoing surveillance of antibiotic resistance in hospital settings. Similar strict interventions should thus be undertaken in the food production industry if we are to successfully contain their clonal dissemination in the food chain.

The detection of the international *K. pneumoniae* ST307 in a human isolate is further evidence of the wide and increasing spread of ESBL-producing bacteria in the country. The *K. pneumoniae* ST307 has been recognized as candidate for becoming one of the prevalent high-risk and clinically relevant clones since its worldwide emergence during the last five years (Villa et al., 2017). The *K. pneumoniae* ST307 lineage is generally capsulated, displays higher resistance to complement-mediating killing, has novel virulence arrays and is associated with CTX-M-15 and KPC encoding plasmids (Villa et al., 2017). Accordingly, *Klebsiella pneumoniae* Carbapenemase (KPC)-producing *K. pneumoniae* ST307 carrying a self-transferable plasmid (IncX3-type) was detected among clinical specimens during a nosocomial outbreak in South Korea (Kim et al., 2017) as was KPC-producing *K. pneumoniae* ST307 harboring pKPN-307 plasmid (Villa et al., 2017). In contrast to these reports, the *K. pneumoniae* ST307 detected in our study did not harbor the CTX-M-15 and KPC enzymes nor the IncX plasmid but rather TEM-1A, LEN12, OXA-9 and SHV-134 as β-lactamases, and colRNAI, IncR and FIA (pBK30683) plasmid incompatibility groups, suggesting a different phylogenetic evolution. The ability of this clonal lineage to acquire novel genetic features may contribute to its increased persistence in the environment and highlights its potential public health threat.

Only *K. pneumoniae* ST11 and ST15 were detected from clinical samples in Yaoundé, Cameroon to date. The detection of two new genome sequences *K. pneumoniae* ST2958 and ST2959 in pigs with different antibiotic resistance profiles and genes, improve our understanding and scope of the molecular epidemiology and evolution of resistant bacteria in the country. The emergence of these ESBL-producing *K. pneumoniae* in pigs and exposed workers within and between abattoirs in Cameroon is of great significance as it confirms their active clonal dissemination via direct contact, and suggests their indirect spread throughout the food chain in the country. These findings further suggest that pigs, pork, and abattoir workers represent a potential reservoir and source of foodborne ESBL-producing *K. pneumoniae* infections in Cameroon and reinforce the crucial importance of implementing appropriate food safety measures and promoting rational antibiotic use.

All isolates except HH510E2I (MIC ≥ 4 μg/ml) were susceptible to ciprofloxacin (MIC ≤ 0.5 μg/ml) suggesting that they might not contain gene encoding for resistance to fluoroquinolones, but *oqx*A and *oqx*B were detected in all isolates. Similarly, although not expressing phenotypic resistance to gentamicin (MIC ≤ 1 μg/ml), amikacin (MIC ≤ 2 μg/ml) and ciprofloxacin (MIC ≤ 0.25 μg/ml), the pig and human isolates, PN030E4 and HN523E1II, harbored *str*A and *str*B encoding for resistance to aminoglycoside together with the fluoroquinolone resistance genes *oqx*A, *oqx*B and *Qnr*B1. This finding thus indicate that these genes might be “silent” in ESBL-producing *K. pneumoniae* circulating in pigs and humans in Cameroon, and could likely be triggered in response to the selective pressure of widespread antibiotic use.

FosA gene a glutathione *S*-transferase that causes enzymatic inactivation of and resistance to fosfomycin, was detected in six out of seven isolates in our study with 100% homology. Fosfomycin is a broad-spectrum antibiotic used extensively in Europe and Africa for treatment of uncomplicated urinary tract infections (Xu et al., 2011). It is receiving renewed interest globally as a therapeutic option for the treatment of infections caused by carbapenem-resistant *Enterobacteriaceae*. Our finding of chromosomal fosA gene in 85.71% our isolates concurs with a report which revealed that several Gram-negative species including *K. pneumoniae*, *K. oxytoca*, *Enterobacter cloacae*, *Enterobacter aerogenes*, *Serratia marcescens*, carry *fos*A gene on their chromosome and are species with intrinsic reduced susceptibility or resistance to fosfomycin (Ito et al., 2017). The widely distribution of *fos*A gene in our study further suggests that *K. pneumoniae* could serve as a reservoir for this gene and facilitate its dissemination to species lacking *fos*A such as *E. coli* in the country. Although the contextualization of this finding is difficult due to the scarcity of molecular epidemiological studies in Africa, this finding concurs with several studies from Asian (Wachino et al., 2010; Hou et al., 2013; Chan et al., 2014) and European countries (Benzerara et al., 2017) which showed the presence of multiple lineages of fosA producing ESBL-*E. coli.* Any interest in repurposing use of old antibiotics should thus be considered with caution and in light of existing environmental reservoirs of resistance genes.

The CGview analyses showed that major parts of the *K. pneumoniae* ST14 (HN523E1II), are present within the reference genome KPN528 (CP020853) (**Figure 2**). However, some discrepancies in both identity and coverage were observed, as part of the rings are lighter colored or missing, implicating the presence of other genetic elements or independent evolution of this isolate. This suggests that closure of our genomes would be essential to decipher and understand the evolutionary biology of ESBL-producing *K. pneumoniae*. Besides, investigation of the resistance genes and comparison along with the phage-associated regions harbored by our isolates, did not reveal the presence of a prophage-encoding resistance genes. This suggests that prophages are unlikely to act as vectors for the dissemination of resistance within our isolates. Nonetheless, the acquisition of MGEs including the plasmids and bacteriophages contribute to the phenotypic and genetic plasticity of their bacterial host and can act as vector for the transfer of resistance determinants and virulence factors leading to increased prevalence. Routine screening for ESBL-producing *K. pneumoniae* colonization in food animals, exposed workers, farms, abattoirs and food products is thus essential for its effective containment.

Even though our findings may not be extrapolated to the overall situation of the country, it is noteworthy that these highlight a serious food safety threat as the study took place in the main abattoirs of the country’s capital. Further high-resolution genotyping studies of ESBL-producing *K. pneumoniae* collected over larger temporal and spatial scales are required to better understand the evolution, molecular epidemiology, and transmission dynamics of these resistant isolates.

## Conclusion

Our study shows that ESBL-producing *K. pneumoniae* is actively disseminating in pigs and occupationally exposed workers in Cameroonian abattoirs and is probably under-estimated considering the absence of molecular epidemiological studies. It underlines the existence of a potential unheeded food safety and public health threat associated with these resistant strains in the country especially if they spread to susceptible people such as immunocompromised. Ongoing efforts and further well-designed epidemiological studies to understand the epidemiology, transmission dynamics and pathways, risk factors and public health implications associated with the food animal reservoir of ESBL-producing bacteria are essential to inform effective interventions for their containment.

## Author Contributions

LF co-conceptualized the study, undertook sample collection, microbiological laboratory and data analyses, prepared tables and figures, interpreted results, contributed to bioinformatics analysis, and drafted the manuscript. RF undertook sample collection, microbiological laboratory analyses, contributed bioinformatics analysis and vetting of the results. MA undertook bioinformatics analyses. AI performed WGS analysis. CD took part in the design of the study, provided material, equipment and reagents, undertook critical revision of the manuscript and coordinated the field implementation. SE co-conceptualized the study and undertook critical revision of the manuscript. All authors read and approved the final manuscript.

## Conflict of Interest Statement

SE is a member of the Global Respiratory Infection Partnership sponsored by an unrestricted educational grant from Reckitt and Benckiser. The other authors declare that the research was conducted in the absence of any commercial or financial relationships that could be construed as a potential conflict of interest.

## Footnotes

1 http://rast.nmpdr.org

2 http://bigsdb.pasteur.fr/klebsiella/klebsiella.html

3 http://www.microbesonline.org/fasttree/
